# Building a Sea Urchin on Shifting Sands

**DOI:** 10.1371/journal.pbio.1001690

**Published:** 2013-10-29

**Authors:** Roland G. Roberts

**Affiliations:** Public Library of Science, Cambridge, United Kingdom

[Fig pbio-1001690-g001]Constructing a multicellular organism from scratch is a staggeringly challenging task. It seems even more miraculous when you consider that it's actually entirely orchestrated by a few thousand genes and their protein products, acting (largely autonomously) within each one of the rapidly dividing cells. The most crucial of these genes and proteins regulate each other in intricately interconnected ways as part of a gene regulatory network, setting up the structure and physiology of the critter's body.

**Figure pbio-1001690-g001:**
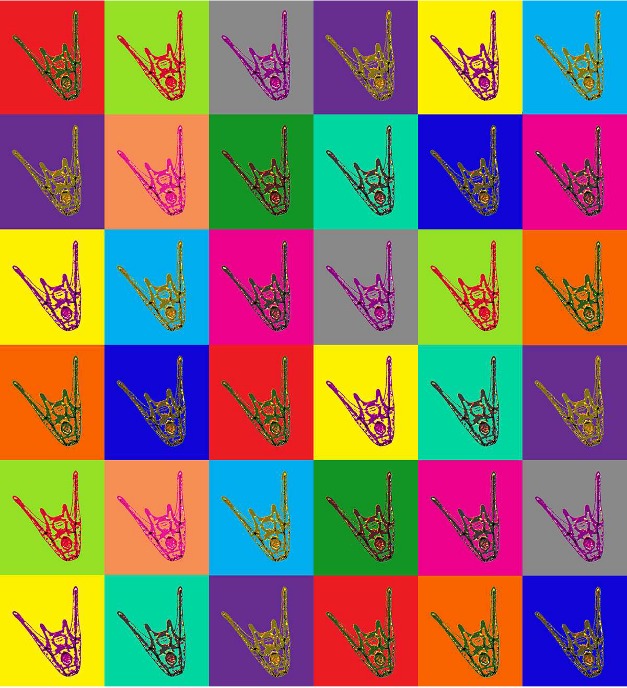
By shifting the distribution of phenotypic variation during development, a gene regulatory network can buffer perturbations yet retain the ability to adapt when the environment changes. *Image credit: Greg Wray and David A. Garfield.*

Already complicated enough, the development of multicellular organisms is also stuck on the horns of a particularly tricky dilemma. On one side, for the short-term perpetuation of an organism, this process must be robust –whatever the challenge, be it genetic mutation or environmental perturbation, the developmental programme has to be able to reliably deliver a viable organism as an end product. On the other side, long-term survival of a species in the face of changing environmental conditions requires that such an organism is able to change, in a genetically determined way, to provide the variability on which natural selection can act. How is it possible to strike this balance between robustness and evolvability?

In a new study, David Garfield, Gregory Wray, and their colleagues examine just this issue, in impressive detail and scale. Their model is the purple sea urchin, a workhorse of developmental biology. Sea urchin embryos develop from single cells to free-swimming larvae over 4 days, and the authors set out to examine how the struggle between robustness and evolvability plays out over this timescale.

To provide the natural genetic variation that acts as a fuel for evolution and a challenge to robustness, the authors collected six female and six male sea urchins from a wild population in the Santa Barbara Channel. They then mated them in every combination to get 36 genetically distinct nurseries of developing embryos. At seven points throughout the ensuing transformation from blob to swimming animal, the authors abstracted hundreds of embryos from each population and monitored the transcript levels of 74 genes from a well-studied developmental network of more than 100 genes.

The design of the experiment meant that the workers could determine whether any effects on gene expression in the embryos had a genetic (as opposed to environmental) cause, by assessing correlations between siblings and attributing influences to either the father (largely genetic) or the mother (both genetic and – via egg provisioning – environmental). Looking at the effects on the expression of single genes, they found that, gratifyingly, natural genetic variation had significant effects on the timing and magnitude of expression of most of the genes examined throughout the course of development. They were then able to scrutinise their data for correlations between genes, enabling them to ask broad questions about regulatory principles.

When two genes are regulated by a common regulator, do they tend to increase or decrease in expression at the same time? Yes, they do, and this correlation tends to increase strikingly as development progresses. Do expression levels of a downstream gene show dependence on levels of an upstream regulator? Yes and no; genes tended to differ substantially in their sensitivity to amounts of the regulator, being either rheostat-like or switch-like. Intriguingly, there seems to be a shift from early switch-like, relatively insensitive regulation to more finely tuneable rheostat-like regulation at later stages of development. The authors speculate that these two distinct regimes reflect a need for robustness (insensitivity to variation) during early development.

The particular gene network examined contains more than 100 interlinked genes involved in determining the axes and cell types of the embryo as well as the structure of the larval skeleton. Sea urchins' skeletons, like ours, are complex, three-dimensional calcified structures. At the end of the 4-day developmental process, the authors took up to 30 larvae from each population, imaged them, and established the three-dimensional relationship between eight skeletal landmarks. The skeleton is a direct product of the gene network under study, and its size and shape have already been shown to influence the fitness of the animal. So by examining the intermediate and ultimate phenotypes of gene expression and skeletal morphology, the authors were able to functionally interrogate the connection between the immediate and ultimate targets of evolutionary selection – fitness and genotype, respectively.

The authors performed a principal component analysis of skeletal data, boiling the large body of information down to three components that captured most of the variation in their sea urchins. Using these parameters to search for significant correlations between shape and the expression of individual genes, Garfield et al. identified eight crucial genes, most of which were already known to play a role in the terminal part of their gene network that's responsible for constructing the skeleton. Naturally occurring variation in genes that play a later and cell-specific role in making the skeleton therefore seems to be responsible for the bulk of the adaptive phenotypic variation in this tissue. There's also a very early, and probably non-genetic, maternal effect on the skeleton that acts independently of the later genes.

Overall, this study builds a comprehensive picture of a large gene network in real, developing animals that are responding to a natural level of genetic variation. What emerges is domination in the early stages of development of switch-like regulatory behaviour that buffers appreciable upstream (cryptic) genetic variation, preventing its manifestation in the phenotype and helping to confer robustness on the system. By contrast, when rheostat-like genes kick in later in development, such as those that determine skeletal variation, variation in gene expression is more tightly and quantitatively coupled to downstream consequences, including skeletal phenotype, and thereby fitness.

Thus, at least for sea urchin development, it seems that the compromise between robustness and evolvability is achieved by separating the fulfilment of these two conflicting requirements in time. The act of making the animal starts with a series of firm binary decisions that lay down a solid developmental foundation, insulating this delicate process from much of the underlying source of variation. What follows is a later creative phase of more sensitive and quantitative regulation, where genetic variation is freer to have its effects and to act as the raw material for evolution.

**Garfield DA, Runcie DE, Babbitt CC, Haygood R, Nielsen WJ, et al. (2013) The Impact of Gene Expression Variation on the Robustness and Evolvability of a Developmental Gene Regulatory Network.** doi:10.1371/journal.pbio.1001696

